# Factors Affecting Psychological Stress in Healthcare Workers with and without Chronic Pain: A Cross-Sectional Study Using Multiple Regression Analysis

**DOI:** 10.3390/medicina55100652

**Published:** 2019-09-27

**Authors:** Yuta Sakamoto, Takeru Oka, Takashi Amari, Satoshi Simo

**Affiliations:** 1 Department of Physical Therapy, Health Science University, Fujikawaguchiko-machi 401-0380, Japan; 2 Department of Rehabilitation Technique, Fuefuki Central Hospital, Fuefuki 406-0032, Japan; okakaookakao16@yahoo.co.jp; 3 Department of Rehabilitation Technique, Ageo Central General Hospital, Ageo 362-8588, Japan; amaritaka@gmail.com; 4 Department of Occupational Therapy, Health Science University; Fujikawaguchiko-machi 401-0380, Japan; sshimo@kenkoudai.ac.jp

**Keywords:** mental health, psychological stress, chronic pain, job stress, medical staff

## Abstract

*Background and Objectives:* Pain affects psychological stress and general health in the working population. However, the factors affecting psychological job stress related to chronic pain are unclear. This study aimed to clarify the structural differences among factors affecting psychological job stress in workers with chronic pain and those without pain. *Materials and Methods:* A stepwise multiple regression analysis revealed the differences in structure between the psychological stress of workers with chronic pain and those with no pain. Psychological job stress by the Brief Job Stress Questionnaire was used as the dependent variable, with psychological state (depression and anxiety), specifically that characteristic of chronic pain (pain catastrophizing); information on the nature of the pain (intensity and duration); and number of years of service as independent variables. Selected independent variables were evaluated for collinearity. *Results:* In the model with psychological stress as a dependent variable (chronic pain: r^2^ = 0.57, F = 41.7, *p* < 0.0001; no-pain: r^2^ = 0.63, F = 26.3, *p* < 0.0001), the difference between the experiences of workers with chronic pain and those with no pain was that chronic pain was associated with depression (Beta = 0.43, *p* < 0.0001) and no pain with anxiety (Beta = 0.34, *p* < 0.0001). In the model with chronic pain-related depression as a dependent variable (r^2^ = 0.62, F = 41.7, *p* < 0.0001), job-life satisfaction (Beta = −0.18, *p* = 0.0017) and magnification (a dimension of pain catastrophizing; Beta = 0.16, *p* < 0.0001) were significant. *Conclusions:* The results of this study suggest that the psychological characteristics of chronic pain, such as depression and magnification, should be considered when evaluating and intervening in the job stress of workers with chronic pain.

## 1. Introduction

A focus on the practical implications of an aging population, driven mainly by an increase in average life expectancy and a decline in birthrate, has become a conspicuous feature of the 21st century. While the focus is usually on increases in this population, the situation affects a number of societal aspects, including the decline of the workforce and its downstream effects [1]. The Japanese population started its decline following the recording of its largest population in 2015; however, the country’s workforce has been on the decline, from 53% in 1995—the country’s largest workforce on record—to 48% in 2015 [2]. The 5% drop in workforce means that the population supporting social security is declining while the number of elderly people receiving social security is increasing, and the burden on workers is increasing. The continued decline in the size of the working population demonstrates the lack of effective interventions to address this issue, even though it has been recognized for more than 20 years. Furthermore, psychosocial and work environments can contribute to health damage caused by mental stress and physical burden, which highlights the socio-economic importance of healthy work conditions [3,4,5]. Thus, it has been shown that improving the health of workers is an important issue not only for individuals but also for society.

Work-related stress is an occupational health challenge and has been identified as a significant contributing factor to burnout syndrome [6], high staff turnover [7], absenteeism [8], and low labor productivity [9]. To counter this, the Japanese government increased its focus on workers’ mental health by launching an occupational health policy, the Stress Check Program, in 2015 [10]. However, since job stress and related psychological states are affected by a number of factors, its uniform evaluation is difficult.

Pain is one of the factors affecting psychological stress in workers, and the cost of pain-related lost productivity ranged from $299 to $335 billion in a large-scale survey using 2008 data on economic loss from pain in the United States; the annual cost of pain is greater than lifestyle-related diseases (heart disease, cancer, diabetes), which are considered to drive large economic losses [11]. Current pain measures often fail to take into account deterioration caused by various factors, including psychological and cognitive aspects, in addition to the sensory experience of noxious stimuli. Since the characteristics of chronic pain are intensified, its assessment should take into account psychological state, such as the presence of anxiety and depression, and level of pain catastrophizing, as evidenced through rumination, helplessness, and magnification of symptoms [12]. Work in the field of chronic pain has shown that psychological states affect pain experience; for example, low social support in the workplace and low job satisfaction are risk factors for back pain [13], while work stress is an independent predictor of chronic neck and shoulder pain [14]. Work-related psychosocial factors are critical in the assessment of health-related quality of life for chronic pain patients [15]. However, thus far the investigation of mental health as it relates to pain is inadequate. We reported on psychological job stress and depression related to chronic pain, as well as psychological stress and anxiety related to the absence of pain, in a previous study on rehabilitation workers [16]. However, a detailed structural explanation for this difference has not yet been provided. The purpose of this cross-sectional study, therefore, was to clarify differences between the factors affecting psychological job stress in hospital workers with chronic pain and in those without pain, using a structural model and multiple regression analysis.

## 2. Materials and Methods

### 2.1. Study Design

This study utilized a cross-sectional design. The sample was recruited from medical staff at a core hospital, and data was collected in February 2018 using questionnaires. The questionnaire survey was distributed to medical staff in all departments who had worked at least 6 months, with the exception of the department in charge of all physicians, which did not provide consent for their staff to participate. Valid questionnaire responses were used to divide the sample into two groups based on duration of pain: a chronic pain group (CPG) and no pain group (NPG). The CPG was defined by continuing or intermittent pain lasting ≥6 months, while the NPG was defined by no pain at the time of the survey and no repetitive pain in the past month. Although previous studies have defined chronic pain as pain lasting longer than 3, or in some cases, 6 months and of at least moderate intensity [17,18,19], our study builds on the notion that chronic pain cannot be evaluated uniformly. We therefore adopted a pain duration of over 6 months as characteristic of chronic pain. 

The procedures were fully explained to participants, in accordance with the Declaration of Helsinki, before the survey, and informed consent was obtained from all subjects prior to participation. The participants were identified by a randomly assigned identifier. This study was conducted with the approval of the Research Ethics Committee of Fuefuki Central Hospital on the 15 August 2017 (Fuefuki, Japan; approval number, 17-4).

### 2.2. Outcome Measure

Job stress was evaluated with the Brief Job Stress Questionnaire (BJSQ), which is used as part of Japanese policy [10]. The BJSQ evaluates job stressors (nine subscales, namely quantity of work, quality of work, physical burden, interpersonal factors, work environment, job control, use of skills, job fit, and job value); psychological stress (five subscales: vigor, irritability, fatigue, feelings of anxiety, and depressed mood); physical stress, based on an evaluation of somatic symptoms; support (three subscales: support from superiors, from colleagues, and from family and friends); and job-life satisfaction. The main outcome was used to evaluate psychological stress [20]. Scores were calculated by summing the outcome scores of the four-point Likert scale (1 = low stress to 4 = high stress); some items were reverse-scored.

One of the chronic pain models [21] suggests pain catastrophizing, anxiety, and depression worsen pain symptoms; additional measures were therefore used to evaluate psychological state and pain-related psychological factors. With this in mind, we used the Hospital Anxiety Depression Scale (HADS), which includes 14 items rated according to a four-point Likert scale and is used to measure anxiety and depression. Physical symptoms are not assessed by HADS, positioning it as a useful measure of chronic pain-related anxiety or depression independent of insomnia or deep symptoms caused by chronic pain. The Pain Catastrophizing Scale (PCS) [22] was used to evaluate pain catastrophizing—a psychological state characteristic of chronic pain. The scale contains 13 items scored on a five-point Likert scale. A subscale assessing rumination, magnification, and helplessness was developed based on the Japanese version of the PCS [23]. Pain characteristics were evaluated as follows: pain intensity was measured using a visual analog scale (VAS) for pain—a subjective evaluation of pain on a scale of 0–100, indicated on a 100 mm line; pain-related disability was measured using the Brief Pain Inventory (BPI), specifically related to the impact of pain on the participant’s life and job; pain duration, measured in number of weeks; and site of pain. 

### 2.3. Statistical Analysis

First, the differences between the two groups in terms of basic characteristics, performance on the five scales of the BJSQ, and psychological state were measured and compared using Mann–Whitney U and chi-square tests. Second, the structural model was verified. Structural model analysis can usually be done using path analysis or structural equation modeling. There are two ways to analyze path models; one of them uses multiple regression analysis. The multiple regression strategy for computing a path analysis employs the ordinary least squares method to calculate the path coefficients [24]. In this approach, the path coefficient is the beta weight of the independent variable, and usually, several multiple regression analyses are used. In this way, an analysis that models multiple independent variables and multiple dependent variable relationships is expressed as multivariate multiple regression [25]. Based on this analysis method, multiple regression analyses were performed twice for each group. A first multiple regression analysis was conducted using psychological stress as dependent variable; analyses were conducted in a stepwise manner according to *p*-value and Bayesian information criterion instead of Akaike’s information criterion, as the emphasis was on model fitness rather than predictive accuracy. Number of years of service, job stress subscales (except that of psychological stress), depression, and anxiety were introduced as independent variables to test for collinearity. The results of the VAS, BPI, and PCS subscales, along with pain duration, were also included into chronic pain as independent variable. Third, another iteration of the multiple regression analysis was performed with the extracted independent variables from the second analysis as dependent variables. The independent variables introduced in the third round were set to the same conditions as for the second analysis, with the exception of the subscale for psychological stress. Furthermore, the validity of model structure in each group was verified through a comparison with the alternative model (AM), which used multiple regression analysis with psychological stress as the independent variable and the dependent variable in a second multiple regression (intragroup-AM). Moreover, the validity of the independent variable in the first multiple regression analysis was verified by comparing the AM, which swapped independent variables between groups (intergroup-AM).

Unless otherwise specified, variables were expressed according to median (lower quartile (LQ) to higher quartile (HQ)). JMP software (version 11.2; SAS Institute, Inc., Cary, NC, USA) was used to conduct the statistical analyses. The indices of multiple regression analysis results were r^2^, F-value, and standardized coefficients (Beta). Beta was used as a value representing the degree of influence of the relevant structure. The critical value for significance was set at *p* value < 0.05.

## 3. Results

**Figure 1:**
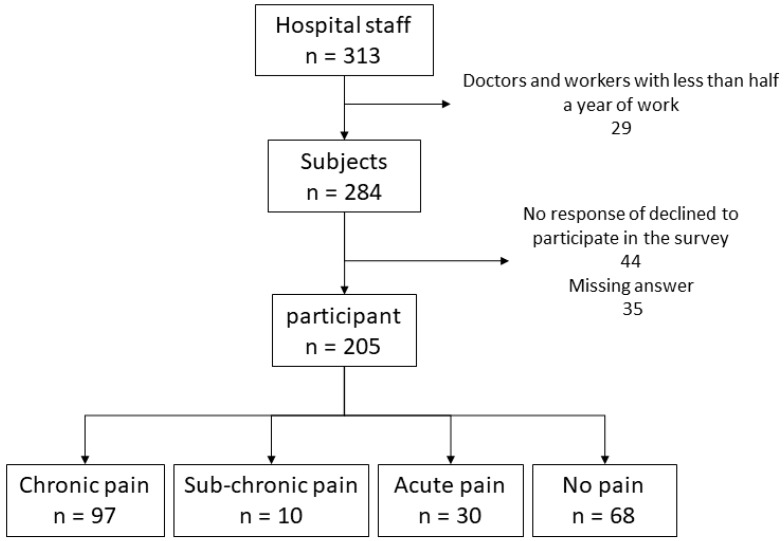
Classification of participants by pain duration. We classified participants according to pain duration. Those included in the no pain group were not experiencing pain at the time of the survey and had not experienced repeated pain in the same site within one month. Acute pain was classified as pain experienced for less than three months, sub-chronic pain was experienced for three to six months, and chronic pain was experienced for over six months. The incidence of chronic pain was the highest, at 47%.

The number of participants is depicted in Figure 1. Out of 284 medical staff members initially contacted, 205 responded with valid answers, resulting in a response rate of 72%. A total of 97 respondents (47%) were included in the CPG, 68 (33%) in the NPG, and 40 participants (20%) could not be included in either group (sub-chronic and acute pain). This division occurred per the criteria discussed previously. The characteristics of the two groups and comparisons between them are shown in Table 1. Mean age in the CPG was 34 years (range: 29.5–41 years). NPG mean age was 34 years (range: 27–37 years; *p* = 0.11). There was no significant difference in gender between the two groups (*p* = 0.083), although the number of females in the CPG was 13% more than in the NPG. The number of years of service showed a similar trend to age, with both the CPG and NPG at 6 years (with ranges of 3–11 and 3–9 years, respectively). There was no difference between office position (*p* = 1) and type of labor contract (*p* = 1). In terms of professional registration or licensing, most participants were nurses (CPG: 47%; NPG: 37%), followed by respondents with no professional registration (CPG: 12%; NPG: 17%). Differences between the two groups were observed for professional registration; however, it was difficult to verify the significance of this difference due to the sample size and research design.

The pain characteristics of the CPG are contained in Table 2. Pain intensity (according to VAS scores) was 48 mm (Interquartile range: 30.5–63.5 mm); BPI-life and BPI-job were 3 (range: 1–5); PCS was 20 (range: 8.5–27); and duration of pain was 208 weeks (range: 91–520). The median for pain persistence experienced as “always” was 22%; 58% of respondents reported that pain affected their work performance; and 13% reported absenteeism. Approximately half had a history of consultation with healthcare providers, and 5% had undergone surgery. The majority of pain sites were reported as, in order, lower back (61%), shoulder (55%), and neck (30%); where multiple pain sites were reported, all were recorded.

The result of the comparison between group outcomes is shown in Table 3. In terms of BJSQ scores, significant differences were found for psychological stress [CPG: 40 (34–47); NPG: 38 (32–42); *p* = 0.039]; physical stress [CPG: 22 (18–25); NPG: 16 (13–19.8); *p* < 0.0001]; and job-life satisfaction [CPG: 6 (5–6); NPG: 6 (5–7); *p* = 0.043]. Differences between job stressors and support were not significant. A significant difference on the scale of psychological states was found for depression [CPG: 6 (3.5–9); NPG: 4.5 (2–8); *p* = 0.037]. Anxiety tended to be higher in the CPG [CPG: 7 (4–9); NPG: 5 (3–8); *p* = 0.054].

**Figure 2:**
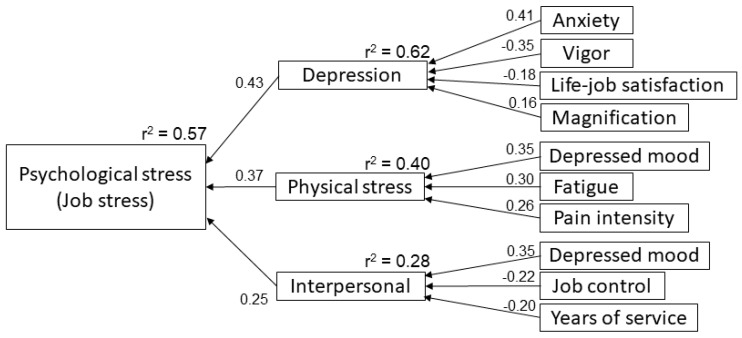
The factor effect model for psychological job stress related to chronic pain. The factor effect model was developed using the results of the stepwise multiple regression analysis using the Bayesian information criterion and *p*-values. The arrow direction moves from the independent variable to the dependent variable, and its numerical value indicates the standardized estimated value. The psychological stress related to chronic pain was most affected by depression, with satisfaction and pain catastrophizing associated through depression.

The factors affecting psychological stress in the CPG, as determined by multiple regression analysis, are shown in Table 4. In the model with psychological stress as a dependent variable (r^2^ = 0.57, F = 41.7, *p* < 0.0001), depression (Beta = 0.43, *p* < 0.0001), physical stress (Beta = 0.37, *p* < 0.0001), and interpersonal factors (Beta = 0.25, *p* = 0.0005) were independent variables. The result of a multiple regression analysis with these independent variables as dependent variables showed that depression as dependent variable (r^2^ = 0.62, F = 41.7, *p* < 0.0001) affected anxiety (Beta = 0.41, *p* < 0.0001), vigor (Beta = −0.35, *p* < 0.0001), job-life satisfaction (Beta = −0.18, *p* = 0.0017), and magnification (a dimension of pain catastrophizing; Beta = 0.16, *p* < 0.0001). Physical stress as dependent variable (r^2^ = 0.40, F = 20.3, *p* < 0.0001) had an impact on depressed mood (Beta = 0.35, *p* = 0.0003), fatigue (Beta = 0.30, *p* = 0.0016), and VAS (Beta = 0.26, *p* = 0.002). Interpersonal factors as dependent variable (r^2^ = 0.28, F = 12.0, *p* < 0.0001) was linked to depressed mood (Beta = 0.35, *p* = 0.0003), job control (Beta = −0.22, *p* = 0.020), and number of years of service (Beta = −0.20, *p* = 0.028). The structure created by multiple regression analysis of CPG data is shown in Figure 2.

In the intragroup-AM of the CPG, the AM with an independent variable of depression (r^2^ = 0.55, F = 28.1, *p* < 0.0001) showed that anxiety (Beta = 0.42, *p* < 0.0001) and vigor (Beta = −0.46, *p* < 0.0001) were higher than Beta in the original model; moreover, job-life satisfaction (Beta = −0.08, *p* = 0.31) and magnification (Beta = −0.012, *p* = 0.88) were lower than Beta in the original model, with no significant difference in *p*-value. The AM with an independent variable of physical stress (r^2^ = 0.89, F =245.5, *p* < 0.0001) showed that depressed mood (Beta = 0.70, *p* < 0.0001) and fatigue (Beta = 0.39, *p* < 0.0001) were higher than Beta in the original model; moreover, pain intensity (Beta = 0.01, *p* = 0.69) was lower than Beta in the original model, with no significant difference in *p*-value. The AM with an independent variable of interpersonal (r^2^ = 0.77, F = 105.7, *p* < 0.0001) showed that depressed mood (Beta = 0.86, *p* < 0.0001) was higher than Beta in the original model; moreover, years of service (Beta = 0.03, *p* = 0.51) and job control (Beta = −0.072, *p* = 0.17) were lower than Beta in the original model, with no significant difference in *p*-value. This result of intragroup p-AM was compared to the original model fit (r^2^) and to the independent variable Beta to determine the appropriate model. The r^2^ of some intragroup-AMs showed a higher increase than in the original model because the intragroup-AM included a subscale of psychological stress as an independent variable. This model was not appropriate because the sum of the subscales was psychological stress. Furthermore, Beta of job-life satisfaction and magnification (a dimension of pain catastrophizing) reduced further than in the original model. This showed that the original model of the CPG was a better model to explain the influential factors than intragroup-AM.

**Figure 3:**
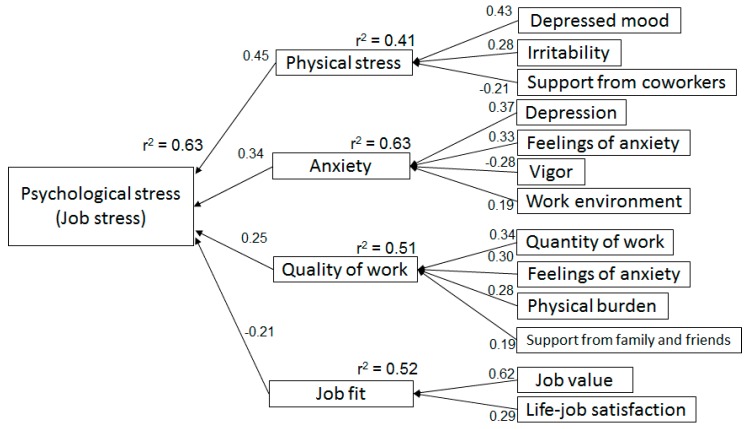
The effect factor model for psychological job stress with no pain. The effect factor model was created through stepwise multiple regression analysis with the Bayesian information criterion and *p*-value. The arrow direction flows from the independent variable to the dependent variable, and its numerical value indicates the standardized estimated value.

The effect factors of psychological stress in the NPG, as determined by multiple regression analysis, are shown Table 5. In the model with psychological stress as a dependent variable (r^2^ = 0.63, F = 26.3, *p* < 0.0001), physical stress (Beta = 0.45, *p* < 0.0001), anxiety (Beta = 0.34, *p* < 0.0001), quality of work (Beta = 0.25, *p* = 0.0002), and job fit (Beta = −0.21, *p* < 0.0001) were independent variables. The result of multiple regression analysis with physical stress as dependent variable (r^2^ = 0.41, F = 26.3, *p* < 0.0001) had depressed mood (Beta = 0.43, *p* < 0.0001), irritability (Beta = 0.28, *p* = 0.0085), and support from coworkers (Beta = −0.21, *p* = 0.036) as independent variables. Where anxiety was the dependent variable (r^2^ = 0.63, F = 26.5, *p* < 0.0001), depression (Beta = 0.37, *p* = 0.0003), feelings of anxiety (Beta = 0.33, *p* = 0.0004), vigor (Beta = −0.14, *p* = 0.0041), and work environment (Beta = 0.19, *p* = 0.015) were affected. Quality of work as dependent variable (r^2^ = 0.51, F = 16.1, *p* < 0.0001) was associated with quantity of work (Beta = 0.34, *p* = 0.0022), feelings of anxiety (Beta = 0.30, *p* = 0.0036), physical burden (Beta = 0.28, *p* = 0.0063), and support from family and friends (Beta = 0.19, *p* = 0.044). Job fit as dependent variable (r^2^ = 0.51, F = 16.1, *p* < 0.0001) was linked to job value (Beta = 0.62, *p* < 0.0001) and job-life satisfaction (Beta = 0.29, *p* = 0.0012). The structure created by multiple regression analysis of the NPG is shown in Figure 3.

In the intragroup-AM of the NPG, the AM with an independent variable of physical stress (r^2^ = 0.89, F = 165.8, *p* < 0.0001) showed that depressed mood (Beta = 0.82, *p* < 0.0001) and irritability (Beta = 0.26, *p* < 0.0001) were higher than Beta in the original model; moreover, support from coworkers (Beta = −0.037, *p* = 0.39) was lower than Beta in the original model, with no significant difference in *p*-value. The AM with an independent variable of anxiety (r^2^ = 0.83, F = 75.4, *p* < 0.0001) showed that feelings of anxiety (Beta = 0.71, *p* < 0.0001) and vigor (Beta = −0.29, *p* < 0.0001) were higher than Beta in the original model; moreover, depression (Beta = 0.036, *p* = 0.55) and work environment (Beta = −0.063, *p* = 0.23) were lower than Beta in the original model, with no significant difference in *p*-value. The AM with an independent variable of quality of work (r^2^ = 0.75, F = 48.4, *p* < 0.0001) showed that feelings of anxiety (Beta = 0.84, *p* < 0.0001) was higher than Beta in the original model; moreover, quantity of work (Beta = 0.032, *p* = 0.47), support from family and friends (Beta = −0.040, *p* = 0.55), and physical burden (Beta = −0.032, *p* = 0.65) were lower than Beta in the original model, with no significant difference in *p*-value. The AM with an independent variable of job fit (r^2^ = 0.11, F = 4.1, *p* = 0.007) showed job-life satisfaction (Beta = −0.33, *p* = 0.007) to be slightly higher than Beta in the original model; moreover, job value (Beta = −0.025, *p* = 0.84) was lower than Beta in the original model, with no significant difference in *p*-value. A comparison of this result with the original model fit (r^2^) and independent variable Beta showed a similarity with the results of the CPG. The r^2^ of some intragroup-AMs of the NPG showed a higher increase than the original model because the intragroup-AM included a subscale of psychological stress as an independent variable. This model was not appropriate because the sum of the subscales was psychological stress. Furthermore, the r^2^ of job fit was reduced further than in the original model. This showed that the original model for the NPG was also a better model to explain the affecting factors than the intragroup-AM.

In the intergroup-AM, the model fit for the CPG was lower than in the first multiple regression model of the CPG (r^2^ = 0.47, F = 20.6, *p* < 0.0001), and this model showed physical stress (Beta = 0.36, *p* < 0.0001), anxiety (Beta = 0.40, *p* < 0.0001), quality of work (Beta = 0.068, *p* = 0.46), and job fit (Beta = −0.13, *p* = 0.10). Moreover, the model fit for the NPG was lower than in the first multiple regression model of the NPG (r^2^ = 0.50, F = 21.2, *p* < 0.0001), and this model showed depression (Beta = 0.35, *p* < 0.0001), physical stress (Beta = 0.56, *p* < 0.0001), interpersonal (Beta = 0.079, *p* = 0.38), and job fit (Beta = −0.13, *p* = 0.10). This result differed from the original model in that the most influential independent variable of the intergroup-AM for the CPG was anxiety, not physical stress, and the main independent variable of the intergroup-AM for the NPG was physical stress, not depression.

## 4. Discussion

### 4.1. Participant Characteristics

This cross-sectional study investigated the factors affecting psychological stress in hospital workers with and without chronic pain. The study had a high response rate (72%) and groups, classified based on the presence or absence of chronic pain, had a median age of 34 years, in line with trends among the labor population. Focusing on the feature of pain in the CPG, 75% of respondents experienced moderate or higher pain, based on the LQ of pain intensity at 30.5 mm on the VAS [26]. However, studies have shown that VAS scores do not coincide with verbal ratings of intensity of chronic pain, and VAS evaluation alone is considered inadequate in the later stages of chronic pain [27]. The median pain duration for CPG participants was 208 weeks—meeting the criteria for chronic pain. Site of pain in the CPG was consistent with the findings of an epidemiological survey in Japan, which also found that the lower back was the most frequent site of chronic pain, followed by the neck and shoulders, respectively [28]. However, the degree of pain-related interference in life and work assessed by two BPI scales had a score of five in the HQ, and more than moderate interference was 25% [29,30]. Based on our results, we can assume that chronic pain affects younger medical staff. Moreover, disability due to chronic pain was not that prevalent.

### 4.2. Validation of Outcome Comparison between Two Groups

The outcome was nonparametric in that there were many mild cases and few severe cases in this study design, and comparison of both groups needed a suitable statistical test. Thus, outcome comparisons were considered along with medians and quartiles. In the comparison of job stress, psychological stress and physical stress were significantly high in the CPG. The difference between psychological stress in the two groups was five points for the HQ and two points for the LQ, suggesting that chronic pain is linked to higher levels of psychological stress. It was considered that the difference of 6 points in the median score for physical stress was caused by the inclusion of pain-related items in the scale. We considered it unlikely that the work-related environment differed among the groups because there was no significant difference in job stressors and support structures. Job-life satisfaction was significantly lower in the CPG, but the degree of difference was small: the HQ in the NPG was only one point higher. In terms of psychological state, the depression levels of the CPG were significantly higher, confirming findings linking high depression to chronic pain in previous studies [31,32]. However, at least approximately 25% of participants in both groups had symptomatic depression, based on their high HADS scores [33]. This suggests that chronic pain is a significant contributor to job stress, in particular psychological and physical stress, regardless of the presence of job stressors or support.

### 4.3. Validation of Structure Model by Multiple Regression Analysis

The multiple regression analysis showed that each group had different factors affecting psychological stress. Our findings positioned depression as an independent variable in the CPG and anxiety as an independent variable in the NPG. In addition, in the multiple regression analysis, CPG-depression had anxiety as an independent variable, and NPG-anxiety had depression as an independent variable. This finding emphasizes earlier findings that depression is important to consider in cases of chronic pain, while anxiety is an important consideration in the absence of pain [16]. However, this finding may also simply involve the effects of participants’ differential abilities to deal with high stress, as CPG scores were significantly higher in the comparison of the two groups.

In the second multiple regression analysis, CPG-depression had job-life satisfaction and magnification (a dimension of pain catastrophizing) as independent variables. There was a difference in job-life satisfaction between the CPG and NPG, and NPG satisfaction had the independent variable of job fit, which is a subscale of job stressors. We interpreted the satisfaction effects of job fit to be appropriate, as the biggest determinant of job satisfaction in epidemiological studies has been found to be an “interesting” job [34]. Furthermore, a job satisfaction and health meta-analysis reported stronger relationships with psychological stress than physical distress [35]. Accordingly, the results of the present study contribute to the understanding of differences in job-life satisfaction affecting job-related psychological stress between people with chronic pain and those with no pain. Our interpretation that magnification increases depression is appropriate; while the differences regarding the PCS subscales were slight, several studies have reported the importance of magnification as a psychological factor [16,36]. Furthermore, pain catastrophizing and depression have additive and adverse effects on the impact of pain, hence both are important assessment measures for chronic pain [31].

The validation process of intragroup-AM reflected that the original model by repeated regression analysis was valid because Beta in the intragroup-AM decreased more than in the original model, except in the independent variable of the psychological stress subscale in both groups. Thus, depression in the CPG and physical stress in the NPG, extracted by the first multiple regression analysis, were the most influential independent variables for each group. This finding was also emphasized in the comparison results between intergroup-AM and the original model. Consequently, our findings are reasonable and suggest that pain catastrophizing, which is a psychological condition that exacerbates chronic pain, can also aggravate psychological job stress by mediating depression.

### 4.4. Highlight of This Study

A highlight of our findings is that workers with chronic pain who had low satisfaction and a high rate of pain catastrophizing show an increase in psychological stress through depression. This is very different to the group with no pain and implies that mental health interventions for medical workers with chronic pain need to consider the psychological conditions characteristic of chronic pain.

The epidemiological investigation of chronic pain in Japan showed that professional workers had the highest chronic pain prevalence of several occupation types [28]. Participants in this study included many professional hospital workers, about half of whom had chronic pain. Thus, hospital workers are responsible for the patient’s health, but they may also themselves be suffering from pain. The health and working environment of hospital workers may be important considerations for ensuring proper quality of medical care. If a mental health improvement program, as a worker health policy, is verified without considering chronic pain, the effect on psychological stress may be overestimated or underestimated. Therefore, the assessment and treatment of chronic pain may also be effective in patients who face high levels of psychological stress.

### 4.5. Limitations

There were some limitations that should be considered in the interpretation of this study. Participants were workers at the medical center but doctors were not included, thus the study cannot explain the situation with doctors. Moreover, the study did not consider the severity of chronic pain or physical and joint function, and the index of satisfaction was one scale for both groups. Furthermore, it is possible that insufficient evaluations contributed to the low r^2^ for physical stress in both groups and in the interspersion of NPG scores (e.g., physical functions that are the origin of physical stress, and pain-related interpersonal factors such as role clarity and recognition) [37]. In addition, it is possible that more specific models could be developed by examining the factors affecting pain and job stress, such as the effort—reward imbalance [38], demand and control [39], and job characteristics [40]. In addition, model verification to burnout, absence, retirement, and chronic pain requires research utilizing different designs, including longitudinal and intervention research. However, the findings of the present study show that the factors affecting job psychological stress differ between workers who experience chronic pain and those who are pain-free, and we consider our findings to be important for future studies of workers’ mental health and chronic pain.

## 5. Conclusions

This cross-sectional study investigated the effect of various factors on psychological stress in hospital workers with chronic pain and those without. Chronic pain is associated with high psychological job stress and physical stress, regardless of the absence or presence of job stressors or support. Furthermore, depression was found to affect psychological job stress in those with chronic pain, while anxiety affected psychological job stress of those with no pain. Moreover, workers with chronic pain, through the effects of job satisfaction and pain catastrophizing, showed increased psychological job stress related to depression. Additionally, the structural model developed for the CPG differed from that developed for the NPG.

Chronic pain is multifaceted in terms of its socio-psychological effects, and this should be considered when evaluating the issue. Based on the results of the present study, it is important to consider the psychological conditions characteristic of chronic pain in order to evaluate and improve the job stress of workers with chronic pain.

## Figures and Tables

**Table 1:** Comparison between groups.

		Chronic Pain Group		No Pain Group		
		n = 97		n = 68		*p*-Value
Age	Median (LQ–HQ) years	34	(29.5–41)	34	(27–37)	0.11
Gender	Female (%)	74	(76)	43	(63)	0.083
Years of service	Median (LQ–HQ) years	6	(3–11)	6	(3–9)	0.26
Office Position	None (%)	81	(84)	57	(84)	1
Labor contract	Regular workers (%)	87	(89)	61	(90)	1
License	Nurse (%)	46	(47)	25	(37)	
	None (%)	12	(12)	12	(17)	
	Physical therapist (%)	9	(9)	4	(6)	
	Medical Social worker (%)	6	(6)	2	(3)	
	Pharmacist (%)	5	(5)	5	(7)	
	Occupational therapist (%)	4	(4)	8	(12)	
	Radiologist (%)	4	(4)	1	(6)	
	Other (%)	12	(13)	11	(12)	

LQ: lower quartile. HQ: higher quartile.

**Table 2:** Pain characteristics of the chronic pain group (CPG).

Evaluation Item		Measured Value
Visual analog scale	median (LQ–HQ) mm	48 (30.5–63.5)
Brief pain inventory-life	median (LQ–HQ)	3 (1–5)
Brief pain inventory-job	median (LQ–HQ)	3 (1–5)
Pain catastrophizing scale	median (LQ–HQ)	20 (8.5–27)
Rumination	median (LQ–HQ)	10 (6–13)
Helplessness	median (LQ–HQ)	5 (2–9)
Magnification	median (LQ–HQ)	4 (1–6)
Duration of pain	median (LQ–HQ) week	208 (91–520)
Recalled trigger ^A^	not reported (%)	51 (55)
Pain persistence ^B^	always (%)	21 (22)
	often (%)	44 (47)
	sometimes (%)	21 (22)
Consultation history	reported (%)	51 (53)
Surgical history	not reported (%)	92 (95)
Influence at work	reported (%)	41 (58)
Absenteeism	not reported (%)	84 (87)
Pain site ^C^	low back (%)	59 (61)
	shoulder (%)	53 (55)
	neck (%)	29 (30)
	head (%)	11 (11)
	knee (%)	10 (10)
	groin (%)	9 (9)
	other (%)	18 (19)

^A^ Missing value was five. ^B^ Missing value was three. ^C^ The site of pain was added respectively. LQ: lower quartile. HQ: higher quartile.

**Table 3:** Comparison between the chronic pain and no pain groups.

	Chronic Pain Group		No Pain Group		*p*-Value
	Median (LQ–HQ)		Median (LQ–HQ)		
BJSQ					
Psychological stress	40	(34–47)	38	(32–42)	0.039 ^A^
Physical stress	22	(18–25)	16	(13–19.8)	*p* < 0.0001
Job stressors	64	(56–68)	63	(57–67)	0.83
Support	26	(23–30)	26	(23–28)	0.39
Job-life satisfaction	6	(5–6)	6	(5–7)	0.043 ^A^
HADS					
Anxiety	7	(4–9)	5	(3–8)	0.054
Depression	6	(3.5–9)	4.5	(2–8)	0.037 ^A^

LQ: lower quartile. HQ: higher quartile. BJSQ: Brief Job Stress Questionnaire. HADS: Hospital Anxiety Depression Scale. ^A^: *p* < 0.05.

**Table 4:** Factors contributing to psychological stress in the chronic pain group.

Dependent Variable	Independent Variable	r^2^	Adj-r^2^	RMSE	F-Value	B	SE	95% CI		β	T Value	*p*-Value
								Lower	Upper			
Psychological stress		0.57	0.56	6.78	41.7							*p* < 0.0001
	1 Depression					1.24	0.22	0.81	1.68	0.43	5.64	*p* < 0.0001
	2 Physical stress					0.74	0.15	0.45	1.04	0.37	5.01	*p* < 0.0001
	3 Interpersonal					1.34	0.37	0.60	2.08	0.25	3.58	0.0005
1 Depression		0.62	0.61	2.20	38.3							*p* < 0.0001
	Anxiety					0.39	0.081	0.25	0.54	0.41	5.26	*p* < 0.0001
	Vigor					−0.56	0.11	−0.78	−0.34	−0.35	−4.97	*p* < 0.0001
	Job-life satisfaction					−0.50	0.20	−0.90	−0.01	−0.18	−2.42	0.017
	Magnification					0.17	0.08	0.01	0.34	0.16	2.14	0.035
2 Physical stress		0.40	0.38	4.00	20.3							*p* < 0.0001
	Depressed mood					0.43	0.11	0.20	0.65	0.35	3.76	0.0003
	Fatigue					0.59	0.18	0.23	0.96	0.30	3.25	0.0016
	Pain intensity (VAS)					0.06	0.02	0.02	0.10	0.26	3.17	0.002
3 Interpersonal		0.28	0.26	1.65	12.0							*p* < 0.0001
	Depressed mood					0.16	0.04	0.08	0.25	0.35	3.77	0.0003
	Job control					−0.28	0.12	−0.52	−0.04	−0.22	−2.36	0.020
	Years of service					−0.06	0.03	−0.11	−0.01	−0.20	−2.23	0.028

Adj-r^2^: adjusted r-square. RMSE: root mean square error. SE: standard error. CI: confidence interval. β: standardized coefficients. VAS: visual analog scale. Note: The analysis’ first step was confirmed by the method of matching factors to variable increase with the Bayesian information criterion and the method of variable increase/decrease with the *p*-value. The second step utilized the least squares method.

**Table 5:** Impact factors for psychological stress in the no pain group.

Dependent Variable	Independent Variable	r^2^	Adj-r^2^	RMSE	F-Value	B	SE	95% CI		β	T Value	*p*-Value
								Lower	Upper			
Psychological stress		0.63	0.60	5.90	26.3							*p* < 0.0001
	1 Physical stress					0.75	0.14	0.48	1.03	0.45	5.45	*p* < 0.0001
	2 Anxiety					0.94	0.24	0.46	1.43	0.34	3.90	0.0002
	3 Quality of work					1.26	0.24	0.45	2.07	0.25	3.10	0.0029
	4 Job fit					−3.19	1.21	−5.61	−0.77	−0.21	−2.64	0.0104
1 Physical stress		0.41	0.38	4.41	15.0							*p* < 0.0001
	Depressed mood					0.59	0.14	0.31	0.86	0.43	4.24	*p* < 0.0001
	Irritability					0.78	0.29	0.21	1.36	0.28	2.72	0.0085
	Support from coworkers					−0.65	0.30	−1.25	−0.04	−0.21	−2.14	0.036
2 Anxiety		0.63	0.60	2.09	26.5							*p* < 0.0001
	Depression					0.31	0.08	0.16	0.46	0.37	4.18	*p* < 0.0001
	Feelings of anxiety					0.53	0.14	0.25	0.82	0.33	3.75	0.0004
	Vigor					−0.43	0.14	−0.72	−0.14	−0.28	−2.98	0.0041
	Work environment					0.92	0.37	0.19	1.65	0.19	2.51	0.015
3 Quality of work		0.51	0.47	1.36	16.1							*p* < 0.0001
	Quantity of work					0.32	0.10	0.12	0.52	0.34	3.20	0.0022
	Feelings of anxiety					0.27	0.09	0.09	0.44	0.30	3.02	0.0036
	Physical burden					0.59	0.21	0.17	1.00	0.28	2.83	0.0063
	Support from family and friends					0.18	0.09	0.004	0.36	0.19	2.05	0.044
4 Job fit		0.52	0.50	0.43	35.1							*p* < 0.0001
	Job value					0.71	0.10	0.51	0.91	0.62	7.15	*p* < 0.0001
	Job-life satisfaction					0.17	0.05	0.07	0.26	0.29	3.38	0.0012

Adj-r^2^: adjusted r-square. RMSE: root mean square error. SE: standard error. CI: confidence interval. Β: standardized coefficients. VAS: visual analog scale. Note: The first step of the analysis was confirmation of matching factors through the variable increase method, using the Bayesian information criterion and variable increase/decrease method by *p*-value. The secondary step used the least squares method.
