# Factors Affecting Psychological Stress in Healthcare Workers with and without Chronic Pain: A Cross-Sectional Study Using Multiple Regression Analysis

**DOI:** 10.3390/medicina56010007

**Published:** 2019-12-24

**Authors:** Yuta Sakamoto, Takeru Oka, Takashi Amari, Satoshi Shimo

**Affiliations:** 1 Department of Physical Therapy, Health Science University, Fujikawaguchiko-machi 401-0380, Japan; 2 Department of Rehabilitation Technique, Fuefuki Central Hospital, Fuefuki 406-0032, Japan; okakaookakao16@yahoo.co.jp; 3 Department of Rehabilitation Technique, Ageo Central General Hospital, Ageo 362-8588, Japan; amaritaka@gmail.com; 4 Department of Occupational Therapy, Health Science University, Fujikawaguchiko-machi 401-0380, Japan; sshimo@kenkoudai.ac.jp

The authors did not realize the error made in the front matter in the proofreading phase. The authors wish to make the following correction to this paper [1]:

An author’s name was originally stated as **Satoshi Simo**, but should be changed to **Satoshi Shimo.**

The authors would like to apologize for any inconvenience caused to the readers by these changes. The manuscript will be updated, and the original copy will remain online on the article webpage.
